# Delineating an extracellular redox-sensitive module in T-type Ca^2+^ channels

**DOI:** 10.1074/jbc.RA120.012668

**Published:** 2020-03-18

**Authors:** Dongyang Huang, Sai Shi, Ce Liang, Xiaoyu Zhang, Xiaona Du, Hailong An, Chris Peers, Hailin Zhang, Nikita Gamper

**Affiliations:** ‡ Department of Pharmacology, Hebei Medical University, Shijiazhuang 050000, China; § Institute of Chinese Integrative Medicine, Hebei Medical University, Shijiazhuang 050000, China; ¶ State Key Laboratory of Reliability and Intelligence of Electrical Equipment, Hebei University of Technology, Tianjin 300401, China; ‖ Key Laboratory of Molecular Biophysics, Hebei Province, Institute of Biophysics, School of Science, Hebei University of Technology, Tianjin 300401, China; ** Faculty of Medicine and Health, University of Leeds, Leeds LS2 9JT, United Kingdom; ‡‡ Faculty of Biological Sciences, University of Leeds, Leeds LS2 9JT, United Kingdom

**Keywords:** calcium channel, oxidation–reduction (redox), zinc, electrophysiology, homology modeling, neuropeptide

## Abstract

T-type (Cav3) Ca^2+^ channels are important regulators of excitability and rhythmic activity of excitable cells. Among other voltage-gated Ca^2+^ channels, Cav3 channels are uniquely sensitive to oxidation and zinc. Using recombinant protein expression in HEK293 cells, patch clamp electrophysiology, site-directed mutagenesis, and homology modeling, we report here that modulation of Cav3.2 by redox agents and zinc is mediated by a unique extracellular module containing a high-affinity metal-binding site formed by the extracellular IS1–IS2 and IS3–IS4 loops of domain I and a cluster of extracellular cysteines in the IS1–IS2 loop. Patch clamp recording of recombinant Cav3.2 currents revealed that two cysteine-modifying agents, sodium (2-sulfonatoethyl) methanethiosulfonate (MTSES) and *N*-ethylmaleimide, as well as a reactive oxygen species–producing neuropeptide, substance P (SP), inhibit Cav3.2 current to similar degrees and that this inhibition is reversed by a reducing agent and a zinc chelator. Pre-application of MTSES prevented further SP-mediated current inhibition. Substitution of the zinc-binding residue His^191^ in Cav3.2 reduced the channel's sensitivity to MTSES, and introduction of the corresponding histidine into Cav3.1 sensitized it to MTSES. Removal of extracellular cysteines from the IS1–IS2 loop of Cav3.2 reduced its sensitivity to MTSES and SP. We hypothesize that oxidative modification of IS1–IS2 loop cysteines induces allosteric changes in the zinc-binding site of Cav3.2 so that it becomes sensitive to ambient zinc.

## Introduction

T-type Ca^2+^ channels (*CACNA1G*, *CACNA1H*, and *CACNA1I* genes; Cav3.1, Cav3.2, and Cav3.3 channel α subunits, respectively) are a family of voltage-gated Ca^2+^ channels with very negative activation thresholds (less than −60 mV) and fast inactivation kinetics (1, 2). The channels are widely distributed in the central and peripheral nervous systems, heart, and vasculature as well as in several types of nonexcitable cells (3, 4). In the central nervous system, T-type channels are highly expressed in dendrites of thalamic and hippocampal neurons, where they amplify subthreshold postsynaptic potentials and facilitate the spread of depolarization to the cell body (5). A negative activation threshold and fast recovery from inactivation make T-type Ca^2+^ channels an important contributor to pacemaker activity in the thalamic, corticothalamic, and other rhythmically active neurons (6, 7). Thalamic T-type currents are enhanced in several rodent models of absence epilepsy; correspondingly, several gain-of-function mutations within *CACNA1* genes are associated with human epilepsies, whereas T-type channel blockers have been shown to suppress seizures and are efficacious in treatment of absent seizures in humans (for a review, see Refs. 4, 8).

In the peripheral nervous system, T-type Ca^2+^ channels (and Cav3.2 in particular) are abundant in small-diameter, capsaicin-sensitive (presumably nociceptive) dorsal root ganglion neurons (9–12), as well as in two distinct types of low-threshold mechanoreceptors innervating skin hair follicles (13). The discovery of a relatively high abundance of T-type Ca^2+^ channels in nociceptors led to establishment of the prominent role of these channels in peripheral nociceptive transmission. Conditional deletion of Cav3.2 (13) or down-regulation in dorsal root ganglia using intrathecal injection of antisense oligonucleotides produced anti-nociceptive effects in rodent pain models of neuropathic and inflammatory pain (14, 15) and reduced the analgesic efficacy of T-type channel blockers (11). Conversely, multiple reports found T-type Ca^2+^ currents or Cav3.2 expression to be increased in chronic pain conditions, such as diabetic neuropathy (14, 16), peripheral nerve injury, or inflammation (12, 17–19).

Given their clear role in epilepsy and pain, regulation of T-type Ca^2+^ channel activity receives intense scrutiny. T-type Ca^2+^ channels are regulated by multiple phosphorylation (20), glycosylation (21, 22), and ubiquitination (12) mechanisms. In addition to these, the Cav3.2 T-type subunit possesses a unique regulatory mode that is targeted by several endogenous regulatory pathways: sensitivity to oxidation and zinc. Thus, Cav3.2 is uniquely sensitive to submicromolar concentrations of extracellular zinc (11, 23–25). The zinc-sensitive module is located extracellularly, involving interaction of the extracellular loops linking the IS1–IS2 and IS3–IS4 transmembrane regions of domain I (23). His^191^, which is absent in Cav3.1 and Cav3.3 subunits, is critical for the high sensitivity of Cav3.2 to zinc and nickel (11, 23, 26). In addition, T-type Ca^2+^ currents recorded in native nociceptive neurons and recombinant Cav3.2 currents are enhanced by reducing agents (DTT or l-cysteine) and inhibited by the oxidizing agent 5,5-dithio-bis(2-nitrobenzoic acid) and hydrogen peroxide (11, 27–30). Moreover, neuropeptide substance P, which induces generation of endogenous reactive oxygen species (ROS)^3^ (31), inhibits endogenous T-type Ca^2+^ currents in primary somatosensory neurons and recombinant Cav3.2 current via an oxidative mechanism (11). Intriguingly, redox modulation of Cav3.2 also depends on His^191^ (11, 24, 32). We have recently shown that oxidative modification of Cav3.2 channels (both recombinant and those endogenously expressed in sensory neurons) induced by substance P enhances channel sensitivity to Zn^2+^ to such an extent that is becomes tonically inhibited by trace amounts of ambient zinc (11). Cav3 channels contain a number of extra- and intracellular cysteines that can be oxidized or reduced, depending on the local redox environment, which, in turn, could affect the conformation of the zinc-binding site. In this study, we combined electrophysiology, site-directed mutagenesis, and computer modeling to delineate the molecular determinants of the unique redox sensitivity of Cav3.2.

## Results

### Cysteine-modifying reagents mimic the effect of redox modulation of Cav3.2 by substance P

**Figure 1.**
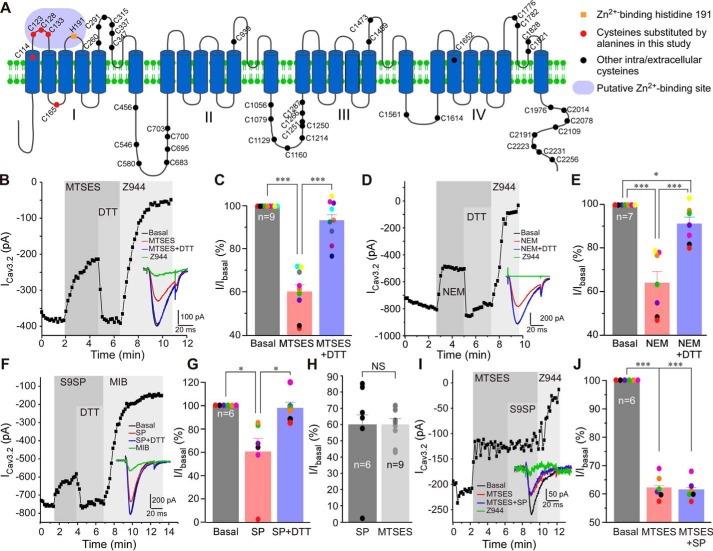
**Cysteine-modifying reagents and substance P inhibit recombinant Cav3.2 currents with a similar mechanism.** *A*, schematic of the Cav3.2 channel; intra- and extracellular cysteines are indicated by *spheres*. Shown in *red* are cysteines mutated in this study. An *orange square* indicates zinc-binding His^191^. The *purple area* indicates the high-affinity zinc-binding site. *B*, example time course of the effects of 2 mm MTSES and 1 mm DTT) applied in the presence of MTSES) on the Ca^2+^ current recorded from HEK293 cells transiently overexpressing Cav3.2 and NK1 receptors using a perforated patch clamp. The selective T-type channel blocker Z944 (1 μm) was applied at the end of the experiment. Plotted are peak Ca^2+^ current amplitudes. Periods of drug application are indicated by *vertical gray bars*. The *inset* shows example current traces. *C*, summary of the effects recorded in the experiments exemplified in *B*. Individual data points are represented by *colored circles*. Paired data points from the same experiment are depicted in the same color. *D*, example time course of an experiment similar to that shown in *A*, but NEM (200 μm) was applied instead of MTSES. *E*, summary of the effects recorded in the experiments exemplified in *D. F*, example time course of an experiment similar to that shown in *B*, but the selective NK1 receptor agonist S9SP (1 μm) was applied instead of MTSES. *G*, summary of these experiments. *H*, comparison of the Cav3.2 current inhibition produced by MTSES and S9SP. *I*, Example time course of the effects of 1 μm S9SP applied after (and in the presence of) MTSES on the recombinant Cav3.2 current. *J*, summary of these experiments. In bar/scatter charts, *asterisks* denote a significant difference between the groups indicated by the *line connectors. NS*, not significant; *, *p* < 0.05; ***, *p* < 0.001 (paired *t* test or one-way ANOVA, as appropriate). *Error bars* represent mean ± S.E. The number of individual recordings is shown within the *bars*.

Neuropeptide substance P (SP) induces production of reactive oxygen species in immune (33) and epithelial (34) cells and sensory neurons (31). In the former cell type, SP inhibits T-type Ca^2+^ current via oxidative modification of Cav3.2 (11). A mechanism of inhibition was proposed to be via enhancement of Zn^2+^ sensitivity of Cav3.2, whereby the oxidized channel is inhibited by nanomolar free Zn^2+^ present in the extracellular milieu (11). However, it is presently unknown how oxidation of Cav3.2 is translated into higher sensitivity to Zn^2+^. Cav3 channels have multiple extra- and intracellular cysteines accessible to redox modulation (Fig. 1*A*). To test whether cysteine modification is necessary for oxidative modulation of Cav3.2, we performed patch clamp experiments testing the effects of the cell-impermeable cysteine-modifying reagent sodium (2-sulfonatoethyl) methanethiosulfonate (MTSES; 2 mm) and the cell-permeable cysteine-modifying reagent *N*-ethylmaleimide (NEM; 200 μm), on the recombinant Cav3.2 overexpressed in HEK293 cells together with the SP receptor NK1 (Fig. 1, *B–J*). MTSES (Fig. 1, *B* and *C*) and NEM (Fig. 1, *D* and *E*) inhibited the Cav3.2 current to similar levels, and inhibition was also similar in amplitude to that produced by the NK1-specific agonist [Sar^9^]-substance P (S9SP; 1 μm; Fig. 1, *F–H*). MTSES, NEM, and SP inhibited peak Cav3.2 current amplitude by 40.1% ± 3.5% (*n* = 9, *p* < 0.001), 36.1% ± 5.4% (*n* = 7, *p* < 0.001), and 40.1% ± 12.3% (*n* = 6, *p* < 0.05), respectively. The reducing agent DTT (1 mm), applied in the presence of MTSES, NEM, or SP (Fig. 1*E*), recovered most of the inhibitory action of the agents, although, in the case of NEM, recovery was incomplete (Fig. 1, *F–H*). Interestingly, when S9SP was applied after MTSES, it produced no further inhibition, suggestive of a common mechanism of action for both agents (Fig. 1, *I* and *J*). MTSES, NEM, or S9SP did not significantly affect the activation or inactivation kinetics of recombinant Cav3.2 (Table S1).

### Ambient zinc and a high-affinity zinc-binding site are necessary for Cav3 channel sensitivity to MTSES

**Figure 2.**
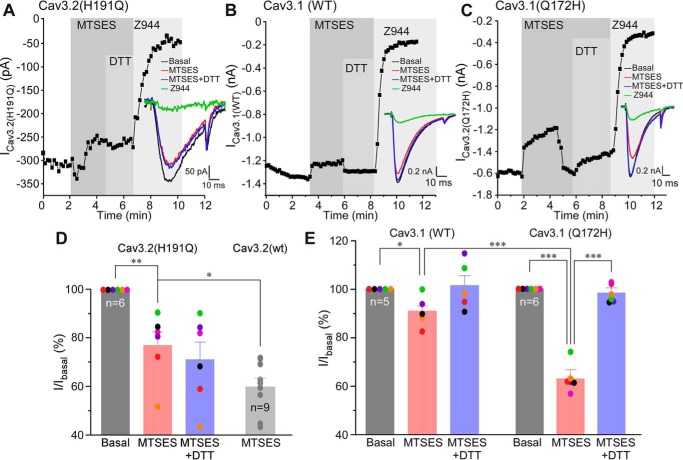
**MTSES-induced inhibition of Cav3 channels requires an intact high-affinity zinc-binding site.** *A–C*, example time courses showing the effects of MTSES (2 mm), DTT (1 mm, applied in the presence of MTSES), and Z944 (1 μm) on the Ca^2+^ current recorded from HEK293 cells transiently overexpressing Cav3.2 H191Q (*A*), WT Cav3.1 (*B*), or Cav3.1 Q172H (*C*). Plotted are peak Ca^2+^ current amplitudes; periods of drug application are indicated by *vertical gray bars*. The *inset* shows example current traces. *D*, summary of the effects recorded in the experiments exemplified in *A*. Individual data points are represented by *colored circles*. Paired data points from the same experiment are depicted in the same color. Also shown is the dataset for MTSES-induced inhibition of the WT Cav3.2 (*gray bar*, *circles*) taken from Fig. 1*C. E*, summary of the effects recorded in the experiments exemplified in *B* and C. In bar charts, *asterisks* denote a significant difference between the groups indicated by the *line connectors*. *, *p* < 0.05; **, *p* < 0.01; ***, *p* < 0.001 (paired or unpaired *t* test or one-way ANOVA, as appropriate). *Error bars* represent mean ± S.E. The number of individual recordings is shown within the *bars*.

It has been shown previously that Cav3.2 with the H191Q mutation in the high-affinity zinc-binding site displayed much lower sensitivity to SP (11). To examine whether His^191^ is important for the effect of MTSES, we tested its effect on the Cav3.2 (H191Q) mutant overexpressed in HEK293 cells together with NK1 receptors (Fig. 2, *A* and *D*). MTSES had only a modest inhibitory effect on Cav3.2 (H191Q) (23.1% ± 5.6%, *n* = 6, *p* < 0.01); inhibition was significantly reduced compared with WT Cav3.2 (Fig. 2*D*, *p* < 0.05). Moreover, MTSES-induced inhibition of Cav3.2 (H191Q) was no longer recoverable with DTT (Fig. 2, *A* and *D*).

Cav3.1 subunit has glutamine instead of histidine in the position equivalent to 191 in Cav3.2 (position 172 in Cav3.1) and, hence, is much less sensitive to zinc (11, 23). Thus, we tested the effect of MTSES on WT Cav3.1 and the Cav3.1 (Q172H) mutant. MTSES produced only very modest inhibition of 9.3% ± 2.8% in WT Cav3.1 (*n* = 5, *p* < 0.05; Fig. 2, *B* and *E*). Strikingly, the MTSES effect on Cav3.1 (Q172H) currents was much stronger (inhibition of 36.9% ± 2.8%, *n* = 6, *p* < 0.001; Fig. 2, *C* and *E*). MTSES inhibition of Cav3.1 (Q172H) was comparable with that of WT Cav3.2 (*cf.* Figs. 2, *C* and *E*, and 1, *B* and *C*). The results further reinforce the hypothesis that oxidative modulation of Cav3 channels depends on the high-affinity zinc-binding site.

**Figure 3.**
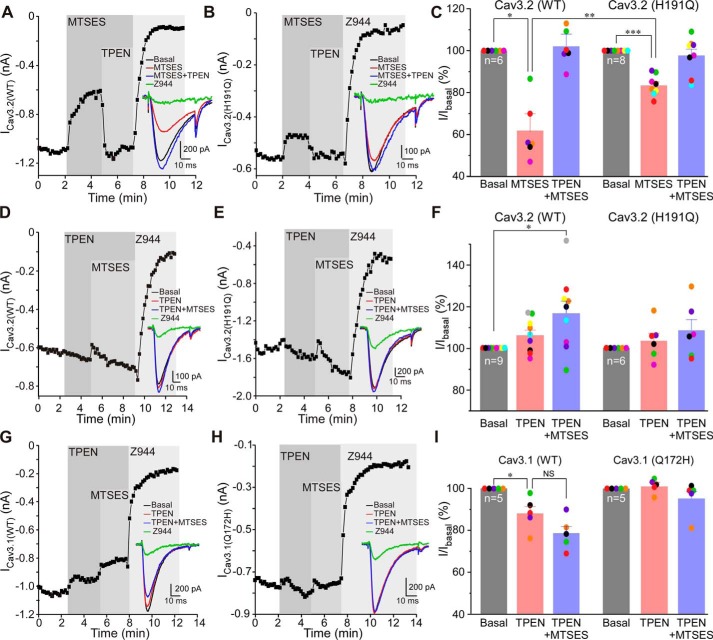
**MTSES-induced inhibition of Cav3 channels is reversed by zinc chelation.** *A* and *B*, example time courses showing the effects of MTSES (2 mm), TPEN (10 μm, applied in the presence of MTSES), and Z944 (1 μm) on the Ca^2+^ currents recorded from HEK293 cells transiently overexpressing WT Cav3.2 (*A*) or Cav3.2 H191Q (*B*). *C*, summary of the effects recorded in experiments exemplified in *A* and *B*. Individual data points are represented by *colored circles*. Paired data points from the same experiment are depicted in the same color. *D* and *E*, experiments similar to those shown in *A* and *B*, but MTSES was applied after (and in the presence of) TPEN. *F*, summary of the effects recorded in the experiments exemplified in *D* and *E. G* and *H*, experiments similar to those shown in *D* and *E*, but WT Cav3.1 (*G*) and Cav3.1 Q172H (*H*) were investigated. *I*, summary of the effects recorded in the experiments exemplified in *G* and *H*. In bar/scatter charts, *asterisks* denote a significant difference between the groups indicated by the *line connectors. NS*, not significant; *, *p* < 0.05; **, *p* < 0.01; ***, *p* < 0.001 (paired or unpaired *t* test or one-way ANOVA, as appropriate). *Error bars* represent mean ± S.E. The number of individual recordings is shown within the *bars*.

To further probe the requirement of zinc for the effect of extracellular cysteine modification on Cav3 channel activity, we applied the zinc chelator *N*,*N*,*N*′, *N*′-tetrakis(2-pyridylmethy1) ethylenediamine (TPEN, 10 μm) before or after MTSES. The experiments presented in Fig. 3 demonstrate that TPEN could totally recover the inhibition induced by MTSES on WT Cav3.2; thus, 2 mm MTSES inhibited the peak Cav3.2 current to 61.2% ± 8.7% of baseline, and TPEN applied in the presence of MTSES recovered the amplitude to 102.4% ± 5.5% of the baseline (*n* = 6; Fig. 3, *A* and *C*). Consistent with previous results, MTSES inhibition of the Cav3.2 (H191Q) mutant was much smaller (inhibition to 83.4% ± 1.7% of baseline, *n* = 8; Fig. 3, *B* and *C*), and this small inhibition was also recovered by TPEN (Fig. 3, *B* and *C*). Application of TPEN on its own (Fig. 3, *D–F*) did not significantly affect the amplitude of WT Cav3.2 or the H191Q mutant (some run-up of the current amplitude was observed in some recordings, but the effect did not reach significance). Importantly, in the presence of TPEN, MTSES was no longer able to inhibit currents produced by either of the channels. Interestingly, under these conditions, we often observed a small and very transient inhibition by MTSES (as can be seen in the examples presented in Fig. 3, *D* and *E*) which then spontaneously recovered. Clearly, binding of zinc to its high-affinity extracellular binding site is necessary for the full inhibitory action of MTSES. We also tested the effect of TPEN on WT Cav3.1 and Cav3.1 (Q172H) mutant currents, and there were no strong effects (Fig. 3, *G–I*). In WT Cav3.1, TPEN produced small inhibition, which is perhaps a nonspecific effect. In the case of the Cav3.1 (Q172H) mutant, TPEN had no significant effect (Fig. 3, *H* and *I*). Importantly, when applied in the presence of TPEN, MTSES had no effect on WT Cav3.1 or the Cav3.1 (Q172H) mutant (Fig. 3, *G–I*). This result was in stark contrast to that shown in Fig. 2, *C* and *E*, where, in the absence of TPEN, MTSES produced strong inhibition of the Cav3.1 (Q172H) mutant, which has its high-affinity zinc-binding site reintroduced. Mutations H191Q (Cav3.2) and Q172H (Cav3.1) did not significantly affect the activation and inactivation kinetics and current densities of the respective channels (Table S2).

In combination, the results presented in Figs. 1–3 point to the following conclusions: cysteine-modifying agents and oxidative modification have similar inhibitory effects on Cav3 channels; these effects require zinc and depend on the high-affinity extracellular zinc-binding site formed by the extracellular loops of domain I, and because MTSES is cell-impermeable, the cysteines mediating the redox inhibition must be located in the extracellular regions of Cav3 channel proteins.

### The cysteines in the IS1–IS2 extracellular loop of T-type Ca^2+^ channels are necessary and sufficient for redox-mediated inhibition of channel activity

**Figure 4.**
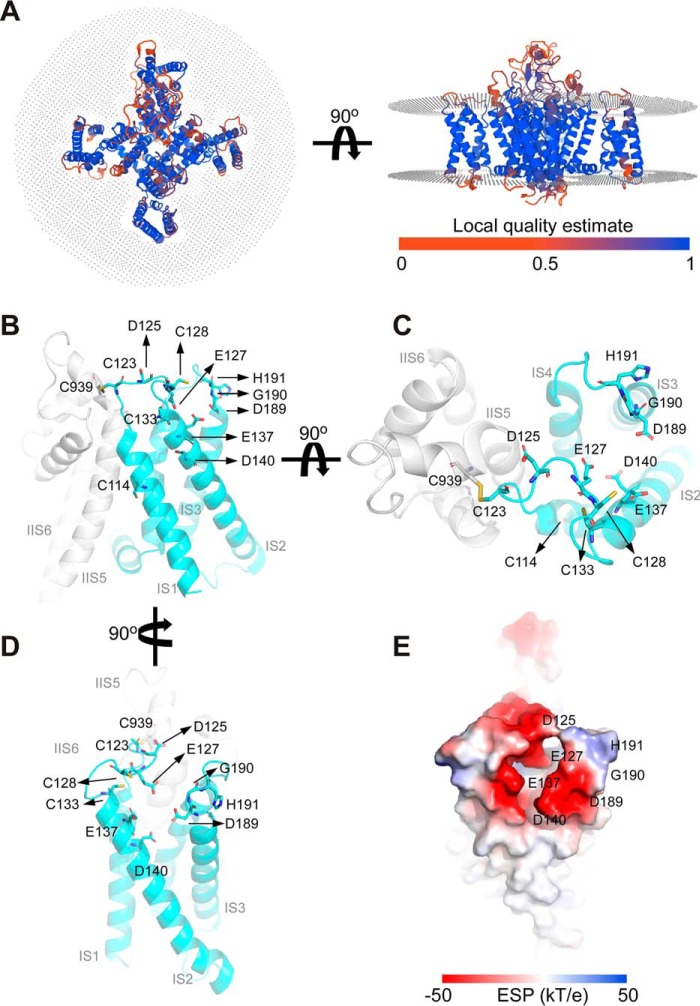
**Homology modeling of the redox/metal-regulatory module in Cav3.2.** The initial structure of the human Cav3.2 channel was obtained by homology modeling (see Fig. S1 for further details) based on the structure of human Cav3.1 (35). *A*, local quality estimate of the model obtained with QMEANBrane. *B–D*, the redox/metal-regulatory module shown at various rotation points (the protein is shown as *ribbons*, and key amino acids are indicated). *E*, electrostatic surface potential of the redox/metal-regulatory module (−50 kT/e to 50 kT/e, in a vacuum).

Earlier studies defined critical residues of the high-affinity metal-binding site of Cav3.2, including an Asp^189^-Gly^190^-His^191^ motif in IS3–S4 and an additional Asp residue in IS2 (23), with His^191^ being a key residue absent in other Cav3 subunits (and any other voltage-gated Ca^2+^ channel α subunits). The high-resolution cryo-EM structure of human Cav3.1 has been solved recently (35). Because Cav3.1 is not highly sensitive to zinc or redox-mediated modulation as a result of absence of the critical histidine residue in its IS3–S4 loop, we obtained the Cav3.2 structure using homology modeling and analyzed the putative metal-binding site (Fig. 4 and Fig. S1). The “local quality” estimate of the model (Fig. 4*A*) shows that the scores of all regions except for a few loop regions are higher than 0.6, which indicates that the overall structure of the model is reliable (Fig. 1*A*).

**Figure 5.**
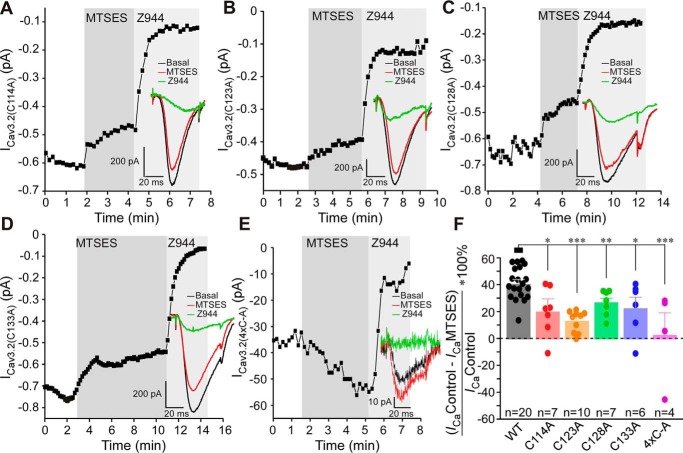
**Extracellular cysteines in the IS1–IS2 loop are necessary for MTSES-mediated inhibition of Cav3.2.** *A–E*, example time courses showing the effects of MTSES (2 mm) and Z944 (1 μm) on the Ca^2+^ current recorded from HEK293 cells transiently overexpressing Cav3.2 C114A (*A*), Cav3.2 C123A (*B*), Cav3.2 C128A (*C*), and Cav3.2 C133A (*D*) or a quadruple mutant with Cys-to-Ala mutations at positions 114, 123, 128, and 133 (*4xC-A*, *E*). *F*, summary of the effects recorded in the experiments exemplified in *A–E*. Individual data points are represented by *circles. Asterisks* denote a significant difference between the groups indicated by the line *connectors*. *, *p* < 0.05; **, *p* < 0.01; ***, *p* < 0.001 (one-way ANOVA). *Error bars* represent mean ± S.E. The number of individual recordings is given below each *bar*.

The putative metal binding site formed by extracellular IS1–IS2 and IS3–IS4 regions is depicted in Fig. 1, *B–E*. The electrostatic surface potential of the Cav3.2 model indicates that the lower potential in the extracellular region comprised of the IS1–IS2 and IS3–IS4 loops is favorable for metal binding (Fig. 1*E*). Moreover, the model suggests that the electrostatic surface potential near Glu^127^ and Glu^137^ is very low, and we suspect that Glu^127^ and Glu^137^ may also contribute to metal binding. Interestingly, four cysteines are located within or near this extracellular region: Cys^114^, Cys^123^, Cys^128^, and Cys^133^ (Fig. 4, *B–D*). Although side chains of several amino acids (*e.g.* methionines, arginines, and aromatic amino acids) can be modified by oxidizers, sulfhydryl groups of cysteines are by far the most susceptible to oxidation protein moieties (36). Cys^123^, Cys^128^, and Cys^133^ have been suggested to be important for modulation of T-type channels by lipoic acid (37) and nitric oxide (38). Thus, we hypothesized that oxidative modification of some or all of these residues may introduce allosteric changes to the metal-binding site, favoring channel inhibition. To test this hypothesis, we substituted these cysteines with alanines and tested the sensitivity of the mutants to MTSES (Fig. 5). All single mutants (C114A, C123A, C128A, and C133A) showed significantly reduced sensitivity to MTSES (Fig. 5, *A–D* and *F*); C123A displayed the least sensitivity (Fig. 5, *D* and *F*). Importantly, the quadruple mutant in which all of the abovementioned cysteines were substituted with alanines was largely insensitive to MTSES (Fig. 5, *E* and *F*). The quadruple mutant expressed very poorly, with current density reduced more than 10-fold compared with WT Cav3.2 (Table S2); hence, only four recordings were produced.

**Figure 6.**
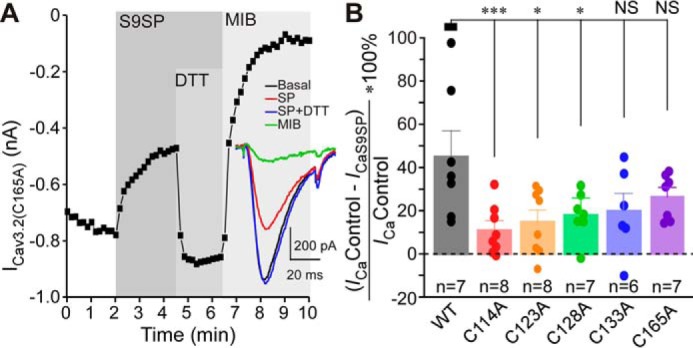
**Extracellular cysteines in the IS1–IS2 loop are necessary for SP-mediated inhibition of Cav3.2.** *A*, example time courses showing the effects of S9SP (1 μm), DTT (1 mm, applied in the presence of S9SP), and Z944 (1 μm) on the Ca^2+^ current recorded from HEK293 cells transiently overexpressing Cav3.2 C165A. *B*, summary of the effects recorded in experiments exemplified in *A* for HEK293 cells overexpressing Cav3.2 C114A, Cav3.2 C123A, Cav3.2 C128A, Cav3.2 C133A, or Cav3.2 C165A. Individual data points are represented by *circles. Asterisks* denote significant difference between the groups indicated by the *line connectors*. *, *p* < 0.05; ***, *p* < 0.001 (one-way ANOVA). *Error bars* represent mean ± S.E. The number of individual recordings is given below each *bar*.

We also tested the sensitivity of individual cysteine mutants to SP (Fig. 6), and again, all four displayed much reduced sensitivity with inhibition by 10 μm S9SP in the range of 10%–20% (compared with 45.1% ± 11.6%, *n* = 7 in the WT Cav3.2). We also tested one intracellular cysteine in the intracellular IS2–IS3 linker, Cys^165^, as we reasoned it could allosterically influence the arrangement of IS1–IS2 and IS3–IS4 loops. However, the C165A mutant still displayed obvious inhibition by SP (26.5% ± 3.9%, *n* = 7), which was not significantly different from SP-induced inhibition of WT Cav3.2 (Fig. 6, *A* and *B*). None of the cysteine mutants used in this study displayed significantly different activation or inactivation kinetics. The current densities of single mutants also did not change significantly compared with WT Cav3.2. The only exception was the quadruple Cys-to-Ala mutant, which displayed much reduced current density (but the kinetics were not significantly affected; Table S2). Taken together, the results presented in Figs. 4–6 strongly suggest that oxidative modification of extracellular cysteines in the IS1–IS2 loop increases sensitivity of Cav3.2 to inhibition by trace amounts of extracellular zinc by inducing conformational changes within the high-affinity metal-binding site of the channel.

## Discussion

Here we report that modulation of T-type Ca^2+^ channels by redox agents and zinc is structurally coupled and depends on the presence of the high-affinity metal-binding site formed by the extracellular IS1–IS2 and IS3–IS4 loops of domain I, as well as on the group of extracellular cysteines present in the IS1–IS2 loop.

A mechanism for convergence of redox- and zinc-dependent modulation of T-type Ca^2+^ channels has been hypothesized in earlier studies, where it was proposed that His^191^ could be subject to a metal-catalyzed oxidation reaction (28, 36). Thus, binding of Zn^2+^ or Zn^2+^-independent metal-catalyzed oxidation of His^191^ could result in similar inhibition of channel activity (24, 28, 39). However, the data presented here suggest an alternative mechanism: oxidative modification of extracellular cysteines in the IS1–IS2 loop may allosterically increase the Cav3 channel inhibition induced by binding of zinc to His^191^. The following observations are in favor of the above hypothesis. The cysteine-modifying reagents MTSES and NEM as well as SP produced similar degrees of Cav3.2 inhibition, which was reversible with the reducing agent DTT (Fig. 1) or the zinc chelator TPEN (Fig. 3); pre-application of MTSES rendered SP ineffective to produce any further current inhibition (Fig. 1, *I* and *J*). Removal of His^191^ from Cav3.2 dramatically reduced inhibition of the channel by MTSES (Fig. 2) or SP (11). Introduction of the corresponding histidine into Cav3.1 induced its sensitivity to MTSES (Fig. 2) and SP (11). Removal of extracellular cysteines from the IS1–IS2 loop of Cav3.2 dramatically reduced the sensitivity of the channel to MTSES (Fig. 5) and SP (Fig. 6); the mutant with the cysteine-less IS1–IS2 loop was found to be resistant to MTSES (Fig. 5, *E* and *F*). Thus, we hypothesize that oxidative modification of extracellular cysteines in the IS1–IS2 loop of Cav3.2 induces allosteric changes in its zinc-binding site so that it becomes sensitive to ambient zinc. This effect is unique to Cav3.2 because other Cav3 subunits lack a critical histidine at positions equivalent to 191 in Cav3.2. Indeed, according to our earlier atomic absorption spectroscopy measurements, total zinc levels in nominally zinc-free laboratory solutions are in the range of 5–10 μm (11). A similar or higher range of zinc concentrations has been reported for human plasma (40). Concentrations of free Zn^2+^ (both *in vitro* and *in vivo*) are likely to be much lower compared with total zinc; our estimate suggested a low nanomolar range (11). This would still be sufficient to have a significant effect on channel activity because the high-affinity zinc-binding site in Cav3.2 has a nanomolar zinc affinity (11).

Activation of NK1 receptors has been shown to generate endogenous ROS production (31, 33, 41), which is a necessary step in NK1-mediated modulation of Cav3.2 (11). However, it is presently unclear how intracellularly generated ROS act upon an extracellular site within the Cav3.2 protein. One intriguing possibility is that, in response to endogenous ROS release, cells could release some redox-active molecules, such as thioredoxin (TRX). Indeed, TRX can be secreted (42). Moreover, it is known to inhibit Cav3.2 channels by interfering with their extracellular zinc-binding site (43). A mechanism of TRPC channel regulation through breakdown of the extracellular disulfide bond by secreted TRX has been reported (42), and it is tempting to hypothesize that a related mechanism could be at play in the case of NK1-mediated modulation of T-type Ca^2+^ channels. However, further investigation is required to decipher this intriguing signaling cascade.

The exact structural consequences of oxidation of IS1–IS2 cysteines have yet to be elucidated. These cysteines may be involved in disulfide bonds or oxidized to cysteine sulfinic (Cys-SO_2_H) or sulfonic (Cys-SO_3_H) acids (44). The Cryo-EM structure of Cav3.1 revealed a disulfide bond between Cys^104^ in IS1–IS2 and Cys^889^ on the IIS5–IIS6 pore loop, which is unique to Cav3 channels (35). This bond was hypothesized to be important for the unique redox sensitivity of T-type Ca^2+^ channels (35). Cys^104^ and Cys^889^ in Cav3.1 correspond to Cys^123^ and Cys^939^ in Cav3.2 (Fig. 1*A*), and our model of Cav3.2 (Fig. 4 and Fig. S1) also predicts a disulfide bond between these residues in Cav3.2, whereas other cysteines in the IS1–IS2 loop do not form disulfide bonds. Interestingly, the C123A Cav3.2 mutant was the least sensitive to MTSES among all cysteine mutants we tested (Fig. 5). Thus, perhaps this covalent bond, linking the high-affinity zinc-binding site to the pore region of the channel, is indeed important for coupling of zinc binding to channel activity and it could be promoted by oxidation. However, because other IS1–IS2 loop cysteines also affect channel sensitivity to MTSES and SP, the Cys^123^-Cys^939^ bond is likely not an exclusive determinant.

Oxidative modification of extracellular cysteines in the IS1–IS2 loop may produce conformational changes within the high-affinity zinc-binding site of the channel that either increases zinc affinity at the binding site or enhances coupling efficiency between zinc binding and channel inhibition. We believe that the latter is more likely to be the case because SP treatment strongly increased the efficacy of zinc-mediated inhibition of Cav3.2 while having no effect on IC_50_ (11). Resolving Cav3.2 structures in the presence and absence of zinc and at different states of extracellular cysteine oxidation will shed the light on the exact mechanism of coupling between these two modulatory mechanisms. Nevertheless, this study clearly demonstrates that the redox and zinc modulation of Cav3.2 is indeed structurally coupled and requires the metal-coordinating histidine in the IS3–IS4 loop and extracellular cysteines in the IS1–IS2 loop.

T-type Ca^2+^ channels are important regulators of excitability and rhythmic activity of excitable cells; the activity of these channels is regulated by multiple physiological signaling pathways, many of which act on the channel targeting its redox/zinc-sensitive module. Examples of these modulatory pathways include nitrous oxide (38, 45), carbon monoxide and thioredoxin (43), hydrogen sulfide (46), α-lipoic acid (37), as well as GABA_B_ receptors (47) and substance P (11). Hence, elucidation of structural background of T-type channel modulation via the redox/zinc-sensitive module reported here sheds new light on the physiological regulation of these channels; moreover, it provides valuable insights into the development of future T-type channel modulators for treatment of excitability disorders, such as epilepsy and pain.

## Experimental procedures

### Cell culture, transfections, cDNA constructs, and chemicals

HEK293 cells were cultured in DMEM supplemented with GlutaMax I, 10% fetal calf serum, penicillin (50 units/ml), and streptomycin (50 μg/ml) in culture flasks in a humidified incubator (37 °C, 5% CO_2_). Cultures were passaged every 2 days (upon reaching 80%–90% confluency); 1 day before transfection, the cells were passaged on glass coverslips (1.3 × 1.3 cm). All cell culture reagents were purchased from Gibco-BRL unless otherwise stated. Human NK1 receptor (GenBank accession number AY462098) cDNA was purchased from the Missouri Science and Technology cDNA Resource Center. Cav3.1 (GenBank accession number AF027984), Cav3.2 (GenBank accession number AF051946), Cav3.1Q172H, and Cav3.2H191Q were kindly provided by Dr. E. Perez-Reyes (University of Virginia). Cav3.2 C114A, Cav3.2 C123A, Cav3.2 C128A, Cav3.2 C133A, Cav3.2 C165A, and Cav3.2 (C114A, C123A, C128A, and C133A) were custom-made by Sangon Biotech. HEK293 cells were transfected using FuGENE HD (Promega) according to the manufacturer's instructions. MTSES and Z944 were from Toronto Research Chemicals. All other chemicals were from Sigma.

### Electrophysiology

Amphotericin B perforated patch clamp recordings were used to record Ca^2+^ currents from transfected HEK293 cells. Recordings were made using a Multiclamp 700B amplifier in combination with pCLAMP 10.4 software (Axon Instruments, Union City, CA) as described previously (11). Offline analysis was performed using Clampfit 10.4 (Molecular Devices). Voltage clamp recordings were sampled at 4 kHz and performed using the Amphotericin B perforated patch clamp method. The standard bath solution contained 150 mm tetraethylammonium-Cl, 2.5 mm CsCl, 2.5 mm CaCl_2_, 10 mm HEPES, 0.5 mm MgCl_2_, and 10 mm glucose (pH 7.4 adjusted with CsOH, 305–310 mosmol/kg). The solutions were applied to the bath chamber using the eight-channel gravity perfusion system VC3–8 (ALA Scientific Instruments) in combination with the local perfusion pencil (04-08-250, AutoMate Scientific; inner diameter, 250 μm) at ∼1 ml/min. The pipette solution contained 155 mm CsCl, 10 mm HEPES, 1 mm EGTA, and 4 mm MgCl_2_ supplemented with amphotericin B (250 μg/ml), pH 7.4-adjusted with CsOH. Patch electrodes were pulled with a horizontal micropipette puller (P-97, Sutter Instruments) and fire-polished. The access resistance was typically within 6–10 megaohm. Cav3 currents were measured by 50-ms square voltage pulses to −40 mV from a holding potential of −90 mV. Series resistance was compensated online by 50%–80%. All recordings were performed at room temperature (∼22 °C).

### Homology modeling

The structural model of the Cav3.2 channel was constructed using the homology modeling server SWISSMODEL (48) and the Cav3.1 channel structure (PDB code 6KZO) (35) as a template. The sequence identity of transmembrane regions of CaV3.1 and CaV3.2 reached 84.27%. The resolution of the Cav3.1 cryo-EM structure is 3.3 Å. The overall root mean square deviation value of atomic positions between the Cav3.2 model and Cav3.1 cryo-EM structure is 0.26 Å (Fig. 1*S*). QMEANBrane was used for reliable local quality estimation of membrane protein models (49). For evaluation of the overall protein structure, the validation server (SAVES v5.0, Institute of Molecular Biology, University of California; RRID: SCR_018219) was used. All molecular visualization and structural diagrams were made using Open-Source PyMOL (RRID:SCR_000305).

### Statistics

All mean data are given as mean ± S.E. Differences between groups were assessed by Student's *t* test (paired or unpaired, as appropriate) or one-way ANOVA with Dunnett's post hoc test. The differences were considered significant at *p* ≤ 0.05. Statistical analyses were performed using Origin 8.6 (OriginLab Corp., Northampton, CA).

### Data availability

All data are available in the main text or the supporting information.

## Author contributions

D. H., C. P., H. Z., and N. G. conceptualization; D. H., S. S., C. L., X. Z., and H. A. investigation; D. H., S. S., C. L., X. Z., H. A., and N. G. methodology; D. H. and N. G. writing-original draft; D. H. and N. G. project administration; S. S. and N. G. visualization; X. D. and N. G. resources; X. D., H. Z., and N. G. supervision; X. D., H. Z., and N. G. funding acquisition; N. G. validation.

## Supplementary Material

Supporting Information

## Footnotes

3 The abbreviations used are:

ROS: reactive oxygen species
SP: substance P
MTSES: sodium (2-sulfonatoethyl) methanethiosulfonate
NEM: *N*-ethylmaleimide
S9SP: [Sar^9^]-substance P
TPEN: *N*,*N*,*N*′,*N*′-tetrakis(2-pyridylmethy1) ethylenediamine
TRX: thioredoxin
cDNA: complementary DNA
ANOVA: analysis of variance
Z944: *N*-[[1-[2-(tert-butylamino)-2-oxoethyl]piperidin-4-yl]methyl]-3-chloro-5-fluorobenzamide.
