# Supplementary material for: Delineating an extracellular redox-sensitive module in T-type Ca2+ channels
Source: J Biol Chem. 2020 Mar 18;295(18):6177–86. doi: 10.1074/jbc.RA120.012668 (PMC7196644; doi:10.1074/jbc.RA120.012668)
Supplement: Supporting Information [file supp_295_18_6177__index.html]

Delineating an extracellular redox-sensitive module in T-type Ca2+ channels — Redox-sensitive module of T-type Ca2+ channels — Delineating an extracellular redox-sensitive module in T-type Ca2+ channels — Redox-sensitive module of T-type Ca2+ channels — Supporting Information 

# Delineating an extracellular redox-sensitive module in T-type Ca2+ channels

## Supporting Information

- Supporting Information (to be published online) - SI figure and tables
