# Supplementary material for: Delineating an extracellular redox-sensitive module in T-type Ca2+ channels
Source: J Biol Chem. 2020 Mar 18;295(18):6177–86. doi: 10.1074/jbc.RA120.012668 (PMC7196644; doi:10.1074/jbc.RA120.012668)
Supplement: Supporting Information [file supp_RA120.012668_158257_2_supp_494239_q7c38y.docx]

**Delineating an extracellular redox-sensitive module in T-type Ca^2+^ channels**

Dongyang Huang, Sai Shi, Ce Liang, Xiaoyu Zhang, Xiaona Du, Hailong An, Chris Peers, Hailin Zhang, Nikita Gamper

**Supporting Information**


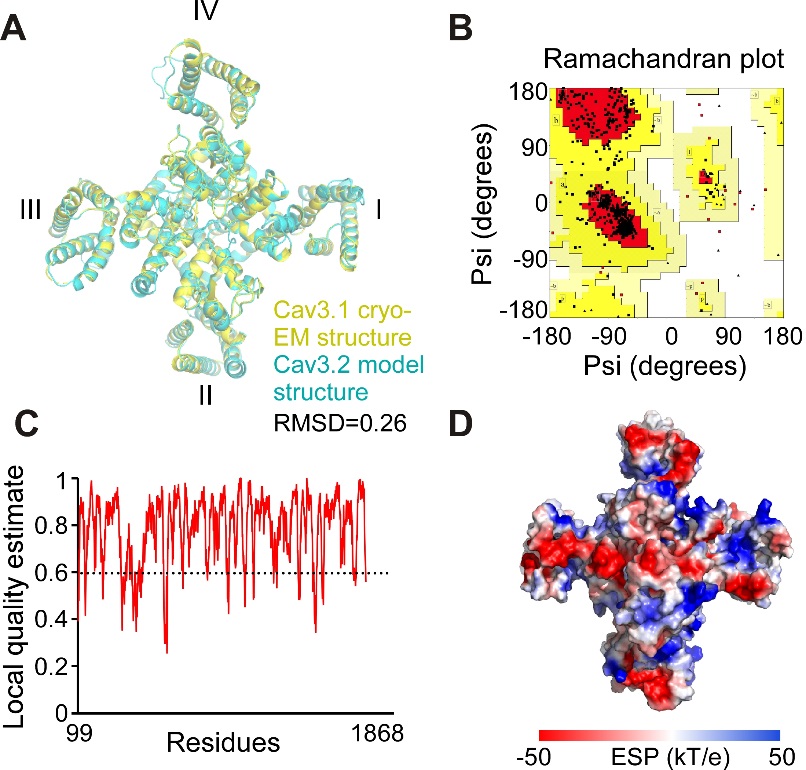


**Figure S1. Model of Cav3.2 channels structure.** **A**, Homology model of Cav3.2 channel (cyan) based on the cryo-EM structure of Cav3.1 (yellow; (35)); the overall root-mean-square deviation of atomic positions (RMSD) value for the model is 0.26. **B**, The Ramachandran plot of the Cav3.2 pore structure shows that the residue occupancy rate of the most favored regions, additional allowed regions, generously allowed regions and disallowed regions were 90.4%, 7.9%, 1.2%, 0.5%, respectively. The occupancy of the residue in the total allowable area reached 99.5%, therefore, the constructed Cav3.2 structure is reliable. C, Local quality estimate for the model (obtained using QMEANBrane). D, Electrostatic surface potential of the model (C; -50 kT/e to 50 kT/e, in vacuum).

**Table S1.** Effects of NK1 receptor agonist [Sar9]-Substance P (S9SP; 1 μ M), Sodium (2-Sulfonatoethyl) methanethiosulfonate (MTSES; 2 mM) and N-ethylmaleimide (NEM; 200 μM) on the activation and inactivation kinetics of Cav3.2.

|  | Basal^*^ | S9SP | Basal^*^ | MTSES | Basal^*^ | NEM |
| --- | --- | --- | --- | --- | --- | --- |
| n | 7 | 7 | 12 | 12 | 7 | 7 |
| τ act. | 4.1±1.2 | 3.6±0.2 | 3.4±0.2 | 3.6±0.2 | 4.5±0.5 | 4.4±0.5 |
| τ inact. | 17.1±1.6 | 17.7±1.4 | 19.2±0.5 | 19.8±1.1 | 17.8±1.2 | 17.0±0.7 |

^*^Paired control (‘basal’) recordings are used for each group.

There was no significant differences from the control values in any group.

**Table S2.** Effect of mutations used in the study on the activation and inactivation kinetics and on the current density of Cav3.2 and Cav3.1.

|  | Cav 3.2 | | | | | | | | Cav 3.1 | |
| --- | --- | --- | --- | --- | --- | --- | --- | --- | --- | --- |
|  | WT | H191Q | C114A | C123A | C128A | C133A | C165A | 4xC-A | WT | Q172H |
| n | 34 | 21 | 15 | 11 | 14 | 12 | 7 | 4 | 10 | 11 |
| τ act. | 3.9±0.3 | 3.4±0.3 | 4.0±0.2 | 3.8±0.3 | 3.8±0.3 | 3.9±0.3 | 3.9±0.6 | 4.3±0.7 | 1.8±0.2 | 1.9±0.1 |
| τ inact. | 18.7±0.8 | 19.0±1.0 | 20.7±0.7 | 19.2±0.9 | 20.4±0.6 | 20.4±0.8 | 20.5±1.1 | 18.1±1.3 | 13.1±0.8 | 13.0±0.5 |
| Peak current density  (pA/pF) | -35.4±3.96 | -27.0±3.4 | -23.1±5.5 | -29.3±3.4 | -28.2±3.8 | -28.0±5.4 | -29.8±5.6 | -3.1±0.9^*^ | -53.3±12.4 | -44.0±7.1 |

Neither of the mutants produced significant effect on the activation or inactivation kinetics of either of the channels. Τau of activation and inactivation among each mutant group of Cav3.2 or Cav3.1 have no significant difference. Current densities were also not significantly affected with the exception of quadruple Cav3.2 mutant with C-to-A substitutions at positions 114, 123, 128 and 133 (4xC-A); peak current density of this mutant was significantly smaller than that of WT Cav3.2 (P<0.001), as well as of all other mutants.
